# Roles of Bulk and Surface Chemistry in the Oxygen Exchange Kinetics and Related Properties of Mixed Conducting Perovskite Oxide Electrodes

**DOI:** 10.3390/ma9100858

**Published:** 2016-10-21

**Authors:** Nicola H. Perry, Tatsumi Ishihara

**Affiliations:** International Institute for Carbon-Neutral Energy Research, Kyushu University, 744 Motooka, Nishi-ku Fukuoka 819-0395, Japan; ishihara@cstf.kyushu-u.ac.jp

**Keywords:** solid oxide fuel cell (SOFC), solid oxide electrolysis cell (SOEC), perovskite, oxygen exchange, defect chemistry, conductivity, chemical expansion, surface chemistry, segregation, strain

## Abstract

Mixed conducting perovskite oxides and related structures serving as electrodes for electrochemical oxygen incorporation and evolution in solid oxide fuel and electrolysis cells, respectively, play a significant role in determining the cell efficiency and lifetime. Desired improvements in catalytic activity for rapid surface oxygen exchange, fast bulk transport (electronic and ionic), and thermo-chemo-mechanical stability of oxygen electrodes will require increased understanding of the impact of both bulk and surface chemistry on these properties. This review highlights selected work at the International Institute for Carbon-Neutral Energy Research (I^2^CNER), Kyushu University, set in the context of work in the broader community, aiming to characterize and understand relationships between bulk and surface composition and oxygen electrode performance. Insights into aspects of bulk point defect chemistry, electronic structure, crystal structure, and cation choice that impact carrier concentrations and mobilities, surface exchange kinetics, and chemical expansion coefficients are emerging. At the same time, an understanding of the relationship between bulk and surface chemistry is being developed that may assist design of electrodes with more robust surface chemistries, e.g., impurity tolerance or limited surface segregation. Ion scattering techniques (e.g., secondary ion mass spectrometry, SIMS, or low energy ion scattering spectroscopy, LEIS) with high surface sensitivity and increasing lateral resolution are proving useful for measuring surface exchange kinetics, diffusivity, and corresponding outer monolayer chemistry of electrodes exposed to typical operating conditions. Beyond consideration of chemical composition, the use of strain and/or a high density of active interfaces also show promise for enhancing performance.

## 1. Introduction: Solid Oxide Fuel and Electrolysis Cells

**Figure 1:**
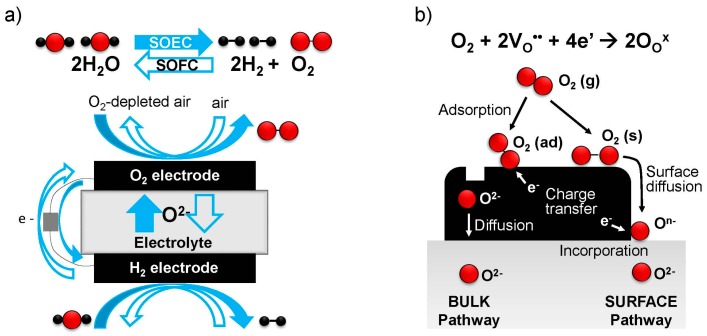
(**a**) Schematic of reversible solid oxide fuel/electrolysis cell; (**b**) two possible oxygen incorporation and reduction pathways in the oxygen electrode (SOFC mode).

Fuel cells directly convert the chemical energy of fuel and oxygen to electrical power with potential for significantly higher efficiency than conventional heat engines [1,2,3,4,5]. Among the various types of fuel cells, solid oxide fuel cells (SOFCs), using oxide ion (or proton) conducting ceramic electrolytes, operate at high temperatures (around 873–1273 K), thus providing the ability to directly use a variety of hydrocarbon fuels with high fuel-to-electricity conversion efficiency (>55%, lower heating value (LHV)) [2]. Moreover, when the waste heat is utilized, in combined heat and power (CHP) applications, they can exceed 85% efficiency [6], unmatched by any other energy conversion technology. Therefore, SOFCs are now attracting much interest for power generation. Components of SOFCs include the cathode (oxygen electrode), anode (fuel electrode), and ionically conducting electrolyte between them; see Figure 1a. Individual SOFC cells are electrically connected in series by interconnects, leaving space for gas channels, to create an SOFC “stack” [1,2,3,4,5]; parallel connection of cells may also be incorporated for stack lifetime considerations. For several decades, Y_2_O_3_ stabilized ZrO_2_ (YSZ) has been the workhorse SOFC electrolyte, because of its reasonably high ionic conductivity, very low electronic conductivity, low cost, and mechanical strength [1,2,3]. Owing to YSZ’s limited ionic conductivity, early SOFCs operated at ~1273 K. While such high temperatures can boost the thermally-activated oxide ion transport, they also result in high cell costs, long start-up and shut down cycles, and unacceptable performance degradation rates. Therefore, at present, improvement in long term stability is an important issue for SOFC development. Contributing factors to power density degradation in SOFCs [7] include (1) poisoning of the electrodes by chemical impurities such as S or Cr; (2) reactions between components; (3) sintering or aggregation; and (4) delamination. Degradation modes specific to the oxygen electrode are discussed in more detail later, but for all of these factors, clarifying the mechanisms and driving forces is a subject of ongoing research [8,9,10,11].

On the other hand, recently, there has also been strong interest in reversible operation of SOFCs, i.e., as “Solid Oxide Electrolysis Cells (SOECs)” from an energy storage point of view. Since the use of renewable energy, such as wind or solar power, is increasing, the generated electrical power may not match demand, because the renewable electric power supply fluctuates. In order to store and average electric power, electrolysis of water into chemical fuel is now attracting interest. There are several types of electrolysis cell, including alkaline, polymer, and high temperature ceramic cells. Here, conversion efficiency from electricity to hydrogen is also important. The conversion efficiency increases in the following order: alkaline (ca. 70%) < polymer (ca. 80%) < high temperature (ca. 90%). In high temperature electrolysis, the Gibbs free energy required for the water splitting reaction, which corresponds to the required applied voltage, decreases with increasing temperature because of the increased heat energy (TdS). Therefore, extremely high efficiency can be achieved in high temperature electrolysis. However, there are still many issues to address for SOECs; in particular, degradation of the cell is more significant compared with SOFCs. At present, the materials used for SOECs are similar to those of SOFCs; however, compared with SOFCs, greater tolerance for severe oxidation and tight gas sealing are required [12,13].

For both types of cell, oxygen electrodes play a highly important role in determining efficiency, and superior catalytic activity, transport properties, and stability are required. In this non-exhaustive review, the role and fundamental properties required of oxygen electrode materials in SOFC and SOEC modes are explained, examples of oxygen electrode chemistries are given, and then investigations into the impact of both bulk and surface chemistry (such as surface segregation) in determining performance and degradation are summarized. The paper primarily focuses on some of the work in progress in this field within the International Institute for Carbon-Neutral Energy Research (WPI-I^2^CNER) at Kyushu University, and additionally, selected work from the broader community is discussed and highlighted.

## 2. Role and Required Properties of Oxygen Electrodes

Oxygen electrodes for solid oxide fuel/electrolysis cells (SOFCs/SOECs) mediate the electrochemical reduction of gaseous oxygen into oxide ions (SOFC cathode) or oxidation of oxide ions into gaseous oxygen (SOEC anode), respectively, at elevated temperatures (typically 873–1273 K). The former process, the oxygen reduction reaction (ORR) at the cathode of SOFCs, is given below in Kroger-Vink (point defect) notation, where the main symbol indicates the species (O = oxygen, e = electron, V = vacancy, and h = hole), superscript indicates the relative charge (′ = negative, × = neutral, · = positive) and subscript indicates lattice site (O = oxygen site, i = interstitial site). The ORR reaction can be written:

(1) $$\frac{1}{2}O_{2}\left(g\right)+2e^{\prime }+V_{O}^{\cdot \cdot }\to O_{O}^{\times }\;\mathrm{or}\;\frac{1}{2}O_{2}\left(g\right)+V_{O}^{\cdot \cdot }\to O_{O}^{\times }+2h^{\cdot }$$

if the electrode conducts oxide ions by an oxygen vacancy mechanism, and

(2) $$\frac{1}{2}O_{2}\left(g\right)+2e^{\prime }+V_{i}^{\times }\to O_{i}^{\prime \prime }\;\mathrm{or}\;\frac{1}{2}O_{2}\left(g\right)+V_{i}^{\times }\to O_{i}^{\prime \prime }+2h^{\cdot }$$

if the electrode conducts oxide ions by an oxygen interstitial mechanism. The former process would be typical of oxygen sub-stoichiometric perovskites with formula ABO_3−δ_, whereas the latter process would be more typical of oxygen super-stoichiometric Ruddlesden-Popper phases A_2_BO_4+δ_, for example. (Often A = La, Sr, Ca, or Ba and the B site is at least partially occupied by a multivalent cation, such as Ni, Fe, Co, Mn, or Cr) The oxygen evolution reaction (OER), taking place at the anode of SOECs, is essentially the reverse reaction.

As these reactions involve gaseous oxygen, electronic charges, and oxide ions, the oxygen electrode should ideally be capable of transporting all of these species. Sometimes this reaction occurs locally at “triple phase boundaries (TPBs)”, where a gas channel (or pore), an electronic conducting phase, and an oxide ion conducting phase contact each other, for example in composite electrodes or in purely electronically conducting electrodes at the interface with the ionically conducting electrolyte. On the other hand, in many mixed ionic and electronic conducting (MIEC) materials, i.e., oxides that can conduct both electronic species and oxygen vacancies or interstitials within their lattice, the reaction may take place over the full surface area of the electrode, depending on the availability of charge carriers. Figure 1b illustrates these different pathways for the ORR [14]—the “bulk pathway” incorporating oxygen over the full electrode surface, and the “surface pathway” where oxygen first diffuses and then reacts at a TPB, the electrode/ electrolyte/gas phase interface.

When the “bulk pathway” is operating, fast bulk chemical diffusion is also essential for efficient transport of oxide ions between the electrode surface and the electrolyte; this factor becomes more important for thicker electrodes and is considered performance-limiting for electrodes with thicknesses significantly above the “critical length”, *L*_c_ = *k*/*D*, where *k* is the (temperature- and oxygen partial pressure (pO_2_)-dependent) surface exchange coefficient, and *D* is the (temperature- and pO_2_-dependent) diffusivity. Alternatively for very thin (or nanostructured, high surface area) electrodes well below the critical length, and often at lower temperatures, the surface exchange process becomes performance-limiting. Therefore, in addition to these transport requirements, the electrode surface must possess catalytic activity to enable the various steps of the surface ORR/OER reactions, such as adsorption/desorption, dissociation/association, charge transfer, and lattice incorporation/excorporation, to progress with low activation energies, and to give a high overall value of *k*.

**Figure 2:**
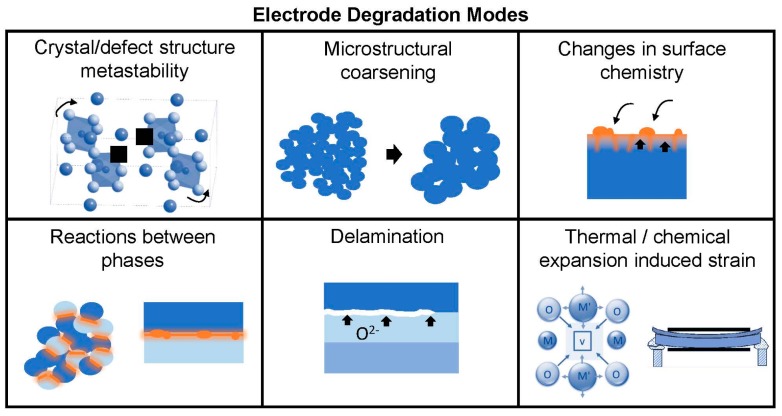
Illustration of degradation modes in oxygen electrode of SOFC/SOEC.

For practical long-term operation, the electrodes should maintain both high *D* and *k* for long periods of time (several years), including start/stop cycles; values of tracer diffusivity, D* ≥ 10^−6^ cm^2^/s and tracer surface exchange coefficient, k* ≥ 10^−4^ cm/s have been suggested [15]. Therefore one final requirement for electrode materials is thermo-chemo-mechanical stability over the range of operating conditions. While higher temperatures are beneficial for enhancing the thermally-activated surface exchange and diffusion processes, the kinetics of performance degradation processes may also be increased at higher temperatures where ions are more mobile. (On the other hand, thermodynamic driving forces for degradation processes may not share this activated temperature dependence). Potential instabilities may arise from: decomposition, ordering, or crystal structure changes of the electrode phase itself if it is metastable in the operating conditions; reactions between composite electrode phases or between electrodes and other cell components; changes in surface chemistry arising from intrinsic surface segregation, extrinsic surface poisoning, or a combination of both; microstructural coarsening; delamination from the electrolyte in SOEC mode when high oxygen chemical potentials occur; and stresses arising from thermal and/or chemical expansion. The latter effect is particular to materials that can change stoichiometry during operation, typically oxygen loss/gain, resulting in localized lattice distortions and macroscopic strain [16]; the corresponding chemical stresses may exceed the strength of the brittle ceramic components [17] and cause mechanical damage [18]. In the case of SOECs, the function of the oxygen electrode is opposite to that of the SOFC cathode, i.e., combination of oxide ions to form gaseous oxygen molecules (reverse of Equations (1) and (2)). Therefore, the high oxygen pressures experienced by the electrode in this mode can lead to unique degradation mechanisms at the oxygen electrode-electrolyte interface, such as chemical strains owing to changes in lattice defect chemistry at high oxygen pressures (e.g., cation vacancies, oxygen interstitials), grain boundary fracture, formation of secondary phases, and delamination [19]. Degradation modes for oxygen electrodes are illustrated in Figure 2.

In this review, the roles of bulk and surface chemistry in impacting the aforementioned electrode properties, particularly the oxygen surface exchange process, will be discussed. The review is limited to oxide ion, rather than proton, conducting electrodes, and focuses on perovskite and perovskite-related structures.

## 3. Example Electrode Chemistries

Traditionally, perovskite-structured oxides (ABO_3_) have been considered the most suitable materials satisfying these requirements for oxygen electrodes, and LaFeO_3_, LaCoO_3_, or LaMnO_3_ doped with Sr or Ca have been widely used. In the early stages of SOFC development, La_0.8_Ca_0.2_MnO_3_ or La_0.6_Sr_0.4_MnO_3_ oxygen electrodes were commonplace; however, because of the low oxide ion conductivity in Mn-based perovskites, the ORR/OER reaction is typically (though not exclusively [20]) limited to the TPB shown in Figure 1, resulting in large cathodic overpotentials (SOFC mode). However, several more complex perovskite oxides have been reported to show very high oxygen surface exchange rates. Baumann et al. [21] compared several Co- and Fe-based perovskites with controlled geometries/morphologies, finding that Ba_0.5_Sr_0.5_Co_0.8_Fe_0.2_O_3−δ_ (BSCF) shows 100 times smaller electrochemical resistance than that of La_0.6_Sr_0.4_Co_0.2_Fe_0.8_O_3−δ_ (LSCF), which is often used for intermediate temperature SOFC cathodes. One reason for such high cathodic activity is the high mixed conductivity, enabling a large reaction area to be available for oxygen incorporation, i.e., the two phase boundary route (“bulk pathway”) in Figure 1. Another highly active oxygen electrode composition is Sm_0.5_Sr_0.5_CoO_3−δ_ which also demonstrates high oxide ion conductivity [22]. However, the most popular composition used for SOFC cathodes still remains La_0.6_Sr_0.4_Co_0.2_Fe_0.8_O_3−δ_ (LSCF), for the reasons of high surface activity, good mixed conductivity, high stability, and moderate cost. Recently, research has shifted to perovskite-related structures such as double perovskites (A_2_B_2_O_6−δ_) and layered Ruddlesden-Popper compounds (A*_n_*_+1_B*_n_*O_3*n*+1_), e.g., *n* = 1 K_2_NiF_4_-related structures. Examples of double perovskites reported to exhibit promising oxygen electrode properties include ABaCo_2_O_6−δ_ (A = Nd, Pr, Sm, Gd) [23,24,25] and Ba_2_Co_2−*x*_B*_x_*O_6−δ_ (B = Sc, Bi) [26], and so not only perovskites but also perovskite-related phases, in particular, highly oxygen deficient perovskite phases, are now attracting much attention as the active oxygen electrode materials for intermediate temperature operation.

**Figure 3:**
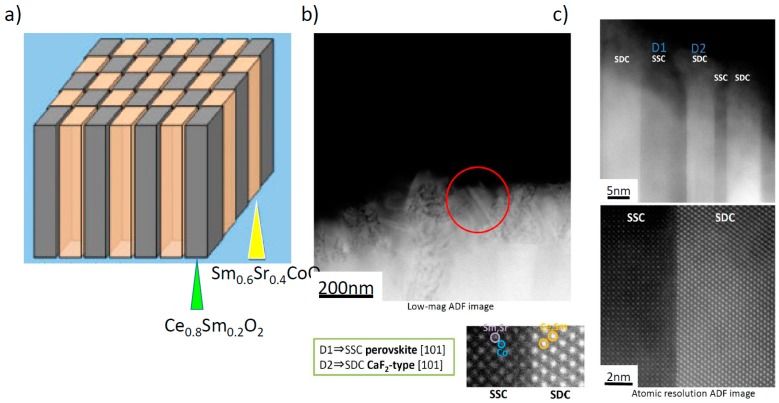
(**a**) Schematic of a double columnar oxygen electrode interlayer containing columns of Ce_0.8_Sm_0.2_O_2_ and Sm_0.6_Sr_0.4_CoO_3_ (**b**) TEM image of the prepared double columnar structure (**c**) High resolution TEM image. Taken with permission from [31] by Ju et al., with one modification: scale bar labels have been enlarged in this version for clarity.

Beyond changing the crystal structure, increasing the mixed conductivity, and increasing the oxygen deficiency, another strategy to improve oxygen electrode performance is nanostructural engineering. Making use of highly active interfaces between phases or within the same phase is one approach; for example Sase et al. [27] reported that the presence of the (La,Sr)_2_CoO_4_ phase on (La,Sr)CoO_3_ electrodes enhances the oxygen exchange reaction rate, and Yildiz et al. [28,29] have proposed explanations for the enhanced ORR activity at the interface between these phases. More recently, grain boundaries in the predominantly electronically conducting LSM have been demonstrated to be active paths for oxygen incorporation, with higher k* and D* than in the grains [30]. Using techniques such as pulsed laser deposition (PLD), it is possible to create thin film electrodes with controlled nano-architectures, exhibiting a high density of interfaces parallel to the direction of oxygen incorporation. In Figure 3, one example of such an approach, named a “double columnar system” or sometimes “vertically aligned nanostructure (VAN)”, is shown schematically [31]. In this structure, nano-sized columns of Sm doped CeO_2_ and Sm_0.6_Sr_0.4_CoO_3_ have been deposited by the PLD method. High resolution transmission electron microscope (TEM) images of the prepared “double columnar system” are shown in Figure 3b,c, where it can be clearly seen that a columnar structure consisting of two different compositions was successfully deposited on the electrolyte substrate.

**Figure 4:**
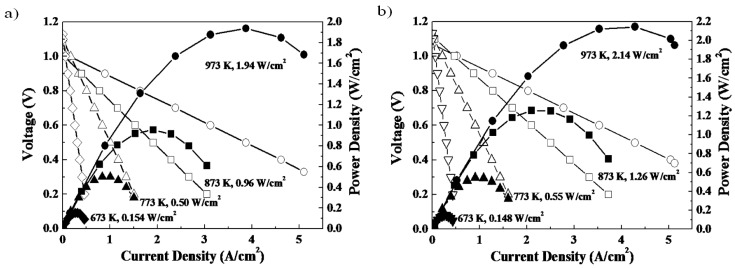
I-V and I-P curves of the cell using (**a**) Sm_0.5_Sr_0.5_CoO_3_ porous electrode fabricated from powder; (**b**) added Sm_0.5_Sr_0.5_CoO_3_/Ce_0.8_Sm_0.2_O_2_ double columnar interlayer between the electrode and electrolyte. LSGM and Ni-Fe (9:1) were used for the electrolyte and fuel electrode, respectively. Taken with permission from [31] by Ju et al.

Figure 4 shows the power generation ability of a SOFC single cell with and without a double columnar interlayer between the electrolyte and porous oxygen electrode [31]. In this cell, a Ni-Fe metallic anode substrate, Sm doped CeO_2_ buffer layer, La_0.9_Sr_0.1_Ga_0.8_Mg_0.2_O_3_ (LSGM) oxide electrolyte (500 μm thickness), and porous Sm_0.5_Sr_0.5_CoO_3_ electrode were used. Although the double columnar layer is nominally dense (i.e., does not presumably have a high surface area), the observed open circuit potential is close to the theoretical one (1.10 V) suggesting its reasonable activity for the ORR process. Obviously, because of the thin LSGM electrolyte film, a high power density close to 2 W/cm^2^ at 973 K is achieved. By comparing the performance of the two cells, it is clear that the power density is increased by using a double columnar structured cathode interlayer. A detailed analysis of the internal resistance suggests that the increased power density can be assigned to the decreased cathodic overpotential. Therefore, control of nano-structure within the oxygen electrode, in this case the increase in two phase boundary area between the mixed conductor and the ionic conductor, can be important for tailoring the ORR/OER activity.

## 4. Role of Bulk Chemistry

With the ultimate goal of optimizing electrode performance by tailoring its chemistry, much work has focused on trying to elucidate relationships between overall (bulk) electrode chemistry, defect chemistry, electronic structure, and electrode properties. Ultimately such descriptors could enable rational design and high throughput search and discovery of superior electrode chemistries. Nonetheless, since many properties must be simultaneously satisfied, and there are usually trade-offs involved, the “ideal” electrode chemistry may depend on particular requirements of an application. Point defect chemistry of bulk electrodes can be inferred from fitting thermogravimetric measurements of oxygen content [32], electrical conductivities [33,34], and/or lattice parameters [35] as a function of temperature and oxygen partial pressure with interrelated mass action expressions of defect generating equations. In thin film electrodes, additional techniques include chemical capacitance and optical absorption measurements [36], which can both indirectly provide information about oxygen content.

### 4.1. Bulk Chemistry Impact on Transport

Oxide ion conductivity, in general, requires both oxygen non-stoichiometry to provide charge carriers ($V_{O}^{\cdot \cdot }$ or O_i_′′) and high mobility of these carriers. Since oxygen ion conduction takes place in the ABO_3_ perovskites by hopping through a triangle made up of two A-site and one B-site cations, the radii (*r*_A_, *r*_B_) and relaxations of these cations during oxygen migration impact the oxide ion mobility [37]. Cherry et al. have noted that a tolerance factor, *t* = (*r*_A_ + *r*_O_)/√2(*r*_B_ + *r*_O_), of 0.81 corresponds to the minimum calculated oxygen migration energy, thought to be due to an optimum balance of relaxations between the A and B cations for this size ratio (i.e., smaller A-site cations and larger B-site cations) [38]. Oxygen vacancy mobility may also be enhanced by more facile reduction of B site cations since transfer of some electron density from oxygen makes it smaller and more able to pass through the A-A-B cation triangle [39,40]; in this sense migration enthalpies may scale with oxygen vacancy formation enthalpies. Tensile strain has also been calculated to decrease the migration barrier for oxygen hopping in perovskites [41]. The presence of cation vacancies and their induced disorder has additionally been suggested to increase the ionic mobility [42].

On the other hand, one must also consider the possibility for trapping of carriers. Oxide ion mobility tends to be decreased when the oxygen defects (e.g., V_O_) are associated with other defects (e.g., acceptors) or otherwise ordered, e.g., by more non-uniform energy landscapes, such as created by cations with different sizes or electronegativities occupying a sublattice. Such effects add a dissociation enthalpy to the migration enthalpy in the conductivity exponential term, which may be overcome at high temperatures where the defects become dissociated or disordered. Larger binding energies of oxygen vacancies and acceptor dopants can contribute to larger overall conductivity activation energies at lower temperatures, and minimizing these binding energies (e.g., as for ${\mathrm{Sr}}_{\mathrm{La}}^{\prime }$-$V_{O}^{\cdot \cdot }$ pairs vs. other dopants in LaGaO_3_ and LaCoO_3_) requires optimizing both elastic strain and electrostatic effects of the dopant [37].

Oxygen non-stoichiometry generally occurs as an ionic compensation mechanism to maintain charge neutrality upon introduction of charged point defects, such as aliovalent dopants, cation vacancies, anti-site defects, or change in valence state of multivalent cations. Many perovskite electrodes therefore intentionally contain acceptor dopants, such as Sr^2+^ on a La^3+^ site, having a relative negative charge, which can be ionically compensated by positively-charged oxygen vacancies:

(3) $$M_{2}O_{3}+2\mathrm{SrO}\overset{{\mathrm{LaMO}}_{3}}{\to }2{\mathrm{Sr}}_{\mathrm{La}}^{\prime }+2M_{M}^{\times }+5O_{O}^{\times }+V_{O}^{\cdot \cdot }$$

Such substitution can also be electronically compensated, in this case resulting in the formation of positively charged holes:

(4) $$\frac{1}{2}O_{2}+M_{2}O_{3}+2\mathrm{SrO}\overset{{\mathrm{LaMO}}_{3}}{\to }2{\mathrm{Sr}}_{\mathrm{La}}^{\prime }+2M_{M}^{\times }+6O_{O}^{\times }+2h^{\cdot }$$

The presence of multivalent cations can support the electronic compensation mechanism and the electronic conductivity, though holes may not necessarily localize exclusively on the multivalent cation. Again, high mobility of the electronic carriers is also important for high electronic conductivity, and significant variety in the extent of charge localization on multivalent cations can be observed in perovskite electrodes. Increasing the concentration of multivalent cation is one way to increase the electronic mobility as the electronic states contributed by these ions broaden or overlap as the concentration increases. For example in the perovskite SrTi_1−*x*_Fe*_x_*O_3−α_, increasing the Fe content from *x* = 0.05 to 0.35 increases the hole mobility by about a factor of 10 [32,33,43]. Similarly, in the perovskite La_0.9_Sr_0.1_Ga_1−*x*_Ni*_x_*O_3−δ_, the conductivity changes from predominantly ionic, to electronic hopping, to apparently metallic (electronic) upon increasing the Ni content to *x* = 0.5 [44]. Aside from acceptor doping, smaller thermal band gaps can increase the intrinsic concentration of electronic carriers.

### 4.2. Bulk Chemistry Impact on Oxygen Surface Exchange

In terms of oxygen surface exchange, the key to improving performance is to identify and improve the rate-limiting step, whether chemisorption/desorption, charge transfer, dissociation/association, or lattice incorporation/excorporation, since the slowest step dominates the reaction kinetics. Complicating factors include the issues that (1) these steps may occur somewhat simultaneously, such as in charge-transfer assisted adsorption or charge-transfer assisted dissociation; (2) many possible detailed reaction pathways exist; and (3) the rate-limiting step can change with operating conditions or over time, and is not always the same across different material systems. Therefore, creating comprehensive “design principles” or chemical descriptors for rapid oxygen surface exchange is not a simple process. Five approaches taken to tackle this challenge are given below with characteristic examples. Please note that these examples are intended as case studies of each approach but are not a comprehensive or exhaustive summary of work performed in this area.

#### 4.2.1. Experimental Studies Seeking to Identify the Rate-Limiting Step for Particular Chemistries under a Limited Range of Conditions

Merkle and Maier have demonstrated a method for homing in on the rate-determining step (RDS) for surface oxygen incorporation by combining spectroscopic information with both equilibrium and non-equilibrium measurements of oxygen exchange kinetics as a function of variables including oxygen partial pressure, temperature, and applied UV light intensity [45]. Their method was applied to Fe-doped SrTiO_3_ single crystals, and the interpretations of the experimental results are enabled by a thorough understanding of the defect chemistry and electronic structure of this composition. Nonetheless, a similar experimental approach may be applied to other materials, bearing in mind that results and interpretations will necessarily be different in materials with different defect chemistry and electronic structures. The general approach is as follows: Intermediate adsorbed oxygen states such as the superoxide O_2_^−^, (more controversial) peroxide O_2_^2−^, and O^−^ radicals may be identified from techniques such as electron paramagnetic resonance (EPR), X-ray or Ultraviolet photoelectron spectroscopy (XPS/UPS), infrared (IR) spectroscopy, and electron energy loss spectroscopy (EELS), giving some insight into observable species present during the reaction. A selective dependence of the reaction kinetics on the intensity of UV light irradiation (with higher energy than the band gap) can indicate the role of electronic carriers in the RDS or a prior step (whether electrons or holes depends on which is the minority carrier that will be relatively more enhanced by exposure to UV light). The measured oxygen partial pressure (pO_2_)-dependence of the reaction rate for small pO_2_ steps (in equilibrium) and of the initial reaction rate for large pO_2_ steps (out of equilibrium) can be compared to what is predicted for each possible mechanism to provide information on the molecularity of oxygen (O_2_*^n^*^−^ vs. O*^n^*^−^) in the RDS and of the number of electrons transferred in or up to that point. For this SrTiO_3_:Fe system their results suggested the presence of O_2_*^n^*^−^ in the RDS with one electron transferred; further, if the bulk and surface defect concentrations share the same pO_2_ dependence, those authors concluded that formation of O_2_^2−^ by electron transfer or dissociation of O_2_^2−^ is the most likely RDS under the measurement conditions. An important point from their work and that of others [46,47] is that the pO_2_ dependence of the reaction rate in equilibrium alone may not be sufficient to identify the fundamental, detailed RDS, since many mechanisms can share the same equilibrium pO_2_ dependence.

#### 4.2.2. Experimental Studies Chemically Varying Point Defect Concentrations or Aspects of Electronic Structure to Identify Controlling Factors for the Surface Exchange Rate for a Particular Chemical System

**Figure 5:**
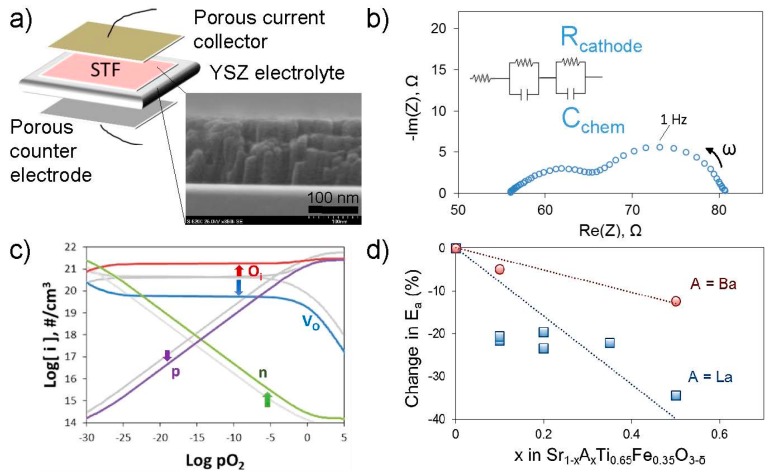
Overview of use of model thin film electrodes with controlled geometries for study of impact of bulk defect chemistry on surface exchange kinetics. (**a**) Schematic of asymmetric cell used for electrochemical impedance measurements of *k*_q_ for thin film (Sr,A)(Ti,Fe)O_3−δ_ electrodes; (**b**) Example impedance spectrum of cell in (**a**) showing equivalent circuit; (**c**) Calculated change in point defect concentrations at 873 K upon La doping, [La] = 0.2 in color vs. [La] = 0 in grey, using model in [34] by Perry et al.; (**d**) Change in activation energy for surface exchange coefficient *k*_q_ for various doping levels; Ba data from [50] by Kim et al. and La data from Perry (unpublished).

Tuller, Jung, Kim, Perry, and co-workers have also applied Sr(Ti,Fe)O_3_ as a model system for probing which aspects of bulk defect chemistry or the related electronic structure are key to fast oxygen surface exchange in this system [34,48,49,50,51]. Isovalent or aliovalent substitution on the A- and B-sites of this perovskite results in changes in oxygen non-stoichiometry and electrical properties that can be measured and modeled to understand the changes in defect concentrations and Fermi level (from the electron and hole concentrations). Dense thin film electrodes deposited by pulsed laser deposition on electrolyte substrates have well-defined geometries enabling comparisons of electrochemically-measured surface exchange kinetics among different compositions (Figure 5).

To date, work along these lines has demonstrated only very weak dependencies of *k*_q_ (electrically or electrochemically driven surface exchange constant) on the ionic and (p-type) electronic conductivities, while the activation energy for *k*_q_ scales with the position of the Fermi level relative to the conduction band (varied by changing the Fe content on the B site) [48]. Since the minority electron concentration in the conduction band is exponentially dependent on the Fermi level position in this strongly p-type material, the result suggested that electron transfer from the electrode conduction band to the adsorbed oxygen was limiting the exchange kinetics. Subsequent work aimed at increasing the minority electron concentration through enhancing reducibility (via Ba substitution on the A-site) [32,50] and through donor doping (via La on the A-site) [34]; in both cases the apparent activation energy for *k*_q_ was lowered. This result was attributed to the rise in bulk Fermi level, though bulk substitution may also change the surface catalytic activity by changing the cations in the outermost surface and sub-surface. Additionally, any expected small changes in absolute values of *k*_q_ are difficult to measure owing to rapid “aging” of the electrodes [34,50], attributed to surface segregation (see also Section 4.4 and figure therein). Effects such as these will be discussed further in the section on surface chemistry. More recent work has aimed at modifying the mobility of the minority electrons in the conduction band, rather than their concentration, e.g., through substitution of Sn for Ti on the B-site [51]. These controlled studies are important for identifying key defects and aspects of electronic structure; however, they do not indicate a detailed reaction mechanism. Additionally, Merkle and Maier have suggested that for this system, charge transfer is only limiting for low Fe contents [52], consistent with the caveat that key factors identified for a particular chemical system may not be broadly applicable; on the other hand, as for the approach in Section 4.2.1, this chemical substitution approach itself can be more broadly applied to various systems.

#### 4.2.3. Compilations of Experimental Data, for a Variety of Electrode Materials, on Surface Exchange Coefficients as a Function of Materials Properties in Order to Find Correlations

In an effort to identify more broadly which defect species or properties are predictors of, or limit, fast surface exchange, some reviews have compiled data from many electrode chemistries to identify correlations. Kilner may have been the first to point out a relationship between the tracer oxygen self-diffusion coefficient (D*) and tracer surface exchange coefficient (k*) both for fluorite-structured and perovskite-structured materials [53]. Log(k*) showed, on average, a linear dependence on log(D*), though with different slopes for the two different structure types. Generally speaking, therefore, electrodes with higher oxygen self-diffusion may also exhibit higher surface exchange coefficients; however, there is considerable scatter in such plots. Wang et al. more recently published a similar plot of log(k*) vs. oxygen ion conductivity for various perovskites, showing again correlation but with wide scatter, particularly at lower ionic conductivity or k* values [54]. Such studies broadly suggest the importance of oxygen vacancy availability for the surface exchange reaction (in oxygen sub-stoichiometric compounds), whether as a site for incorporation or as a donor enabling more facile charge transfer at the surface, assisting chemisorption and/or dissociation. Other early work by Boukamp et al. [55] noted the importance of electronic charge transfer in the surface exchange reaction broadly, comparing electronic conductivities and values of *k* for two fluorite compositions and mixed conducting perovskites in general. Though these data are limited, a plot of log(k*) vs. log(electronic conductivity) also suggests a linear relationship when plotted over a wide enough range. More recently, De Souza introduced an empirical expression for k* which indicated that high concentrations of electronic carriers (electrons and holes) and low concentrations of oxygen vacancies would lead to fast oxygen reactions at the surface, i.e., that donor-doped low band-gap materials or those with oxygen interstitial conduction would be better [56].

#### 4.2.4. Computational Studies of a Limited Range of Chemistries to Identify Key Aspects of Bulk Chemistry and Electronic Structure Vital for the Exchange Reaction

Computational approaches have the advantage of being potentially a more rapid method to investigate structure-property relationships relating to surface reactions with atomistic insight and the disadvantage of being more challenging to apply to high temperature situations of potentially ambiguous surface chemistry. As an example of work in this area, density functional theory (DFT) calculations by Morgan’s group combined with electrochemical measurements in Shao-Horn’s group have suggested a correlation between the calculated bulk O 2p band center (vs. the Fermi level) of the electrode and its measured high temperature surface exchange kinetics (k* or *k*_q_), including the activation energy [57]. Their work has so far encompassed several Ruddlesden-Popper and perovskite compositions. These groups suggested that this aspect of the bulk electronic structure could be a predictive descriptor for rapid surface exchange; on the other hand, a broad set of experimentally measured surface exchange data from the literature did not correlate clearly with the calculated bulk O 2p band centers, possibly due to variations in sample preparation or measurement approach. DFT simulations combined with nudged elastic band calculations have also provided more atomistic insight into the reaction pathway and energetics for oxygen incorporation for selected perovskite and Ruddlesden-Popper phases. Such work is described in detail in the later section on surface chemistry.

#### 4.2.5. Computational Materials-by-Design Approaches to Identify with New Chemistries Predicted High Surface Exchange Rates on the Basis of Previously Identified Descriptors

Once structure-property relationships, i.e., descriptors relating bulk chemistry to defect chemistry and electronic structure and ultimately to surface exchange kinetics, have been identified, the logical next step is to conduct computational searches for chemistries that exhibit this particular descriptor. Searches may make use of databases of both existing and predicted (but not synthesized) stable chemistries. Such studies can be accompanied by high-throughput experimental screening for suitable performance over a wide range of chemistries. Similar approaches have been applied recently to identify candidate perovskite chemistries for thermochemical water splitting [58] and metal alloy anode catalysts for low temperature fuel cells [59]. D. Morgan’s group recently reported screening candidate SOFC cathode materials on the basis of the O 2p band center (for surface exchange kinetics), thermodynamic stability, and band gap (for electronic conductivity) [60].

### 4.3. Bulk Chemistry Impact on Thermo-Chemo-Mechanical Stability

**Figure 6:**
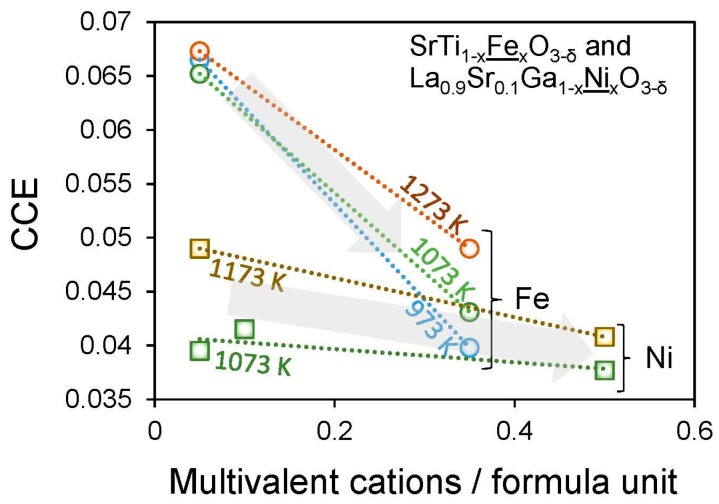
Coefficients of chemical expansion, CCEs, as a function of multivalent cation concentration per formula unit, i.e., *x* in SrTi_1−*x*_Fe*_x_*O_3−δ_ and in La_0.9_Sr_0.1_Ga_1−*x*_Ni*_x_*O_3−δ_ demonstrating correlation between charge delocalization and decreased CCEs. Data from [43,61] by Perry et al.

A number of degradation mechanisms may take place within or around the oxygen electrode over time at elevated temperatures or during start-stop cycles, as described previously in Section 2 and Figure 2. One significant mechanism under investigation is the induction of stresses owing to chemical expansion, when electrode materials undergo changes in oxygen content (Δδ) causing local lattice dilation/contraction and chemical strain (ε_C_) [16]. For example, during the oxygen evolution reaction in sub-stoichiometric materials (reverse of Equation (1)), materials typically expand, as an oxygen vacancy is created and the compensating electronic charge concentration changes. Localization of electrons on multivalent cations (such as creating Ce^3+^ in place of Ce^4+^) drives the expansion. Failure modes including cracking and delamination can result [18], since the stresses generated can be significantly larger than the strength of the materials (particularly if flaws are already present) [17]. Chemical stress mitigation may be achieved through altering operating/start-stop conditions, engineering component morphologies, or changing intrinsic materials properties including the strength, toughness, surface exchange coefficient, oxygen diffusivity, and coefficient of chemical expansion, CCE = ε_C_/Δδ. Perry, Bishop, Marrocchelli and co-workers have been investigating which factors impact CCEs in the perovskite structure [16,35,43,61,62] which is currently the most widely-used structure in oxygen electrodes. Such factors may span many length scales, so a combination of atomistic simulations (density functional theory (DFT), molecular dynamics) with experiments at both the crystal structure (in situ X-ray diffraction, neutron diffraction) and macro-structure levels (thermogravimetric analysis, dilatometry) is applied. To date, some factors that have been identified as significantly impacting CCEs in perovskites include charge localization on cations [43,61], size of the oxygen vacancies [35], temperature [62], and crystal symmetry [61,63]. Of these, charge delocalization may be the most promising approach, as it can be accomplished easily by increasing the concentration of the multivalent cation, which lowers the CCE [43,61] and simultaneously increases both the electronic conductivity and (at least sometimes) the surface exchange kinetics [33,44,49]. On the other hand, such materials with higher concentrations of multivalent cation also typically exhibit larger changes in stoichiometry (Δδ) for a given pO_2_ or temperature change, which also contributes to the chemical strain [43,61]. Figure 6 shows the impact of increasing the multivalent cation, to delocalize charge, on CCEs of two mixed conducting perovskite electrodes.

The effective size of oxygen vacancies is also an important factor, since smaller oxygen vacancies lead to smaller CCEs upon oxygen loss by counteracting some of the cation expansion [64]. A combination of first principles calculations, experimental data, and development of an empirical model recently led to some of the first determinations of the effective size of oxygen vacancies in perovskite oxides [35]; on average the vacancy tends to be about 97% the size of the oxide ion (cf. 72% in the fluorite structure [65]) but with considerable variation among different compositions. Learning how to tailor the oxygen vacancy size via bulk chemistry could enable better control of CCEs in this structure.

### 4.4. Relationship between Bulk and Surface Chemistry

As described above, bulk chemistry is known to impact many oxygen electrode properties, including the oxygen surface exchange kinetics, but since this latter property takes place at the surface, the local chemistry in the outer atomic layers of the electrode should be considered. Surface chemistry can differ from bulk chemistry via effects including surface reconstructions, space charges, modified point defect formation energetics, polarity, segregation, and extrinsic adsorption and reactions forming new phases, but in each case, there is a relationship between the bulk and surface chemistries. (If bulk and surface chemistries were not somehow related, bulk chemical descriptors for fast surface exchange would not exist.) Both experimental and computational approaches are providing insight into this relationship.

**Figure 7:**
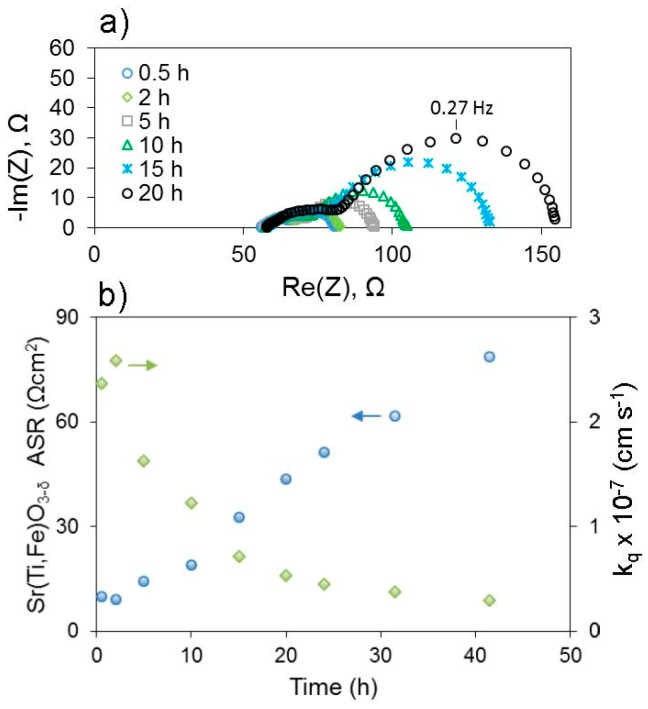
(**a**) Example impedance spectra changes of asymmetric cell with dense thin film (~100 nm thick) SrTi_0.65_Fe_0.35_O_3−δ_ electrode over time at 873 K and 0.21 atm O_2_ by Perry (unpublished; see Figure 5 for cell geometry and equivalent circuit and [34] for more details on the general approach). The rapid increase in area specific resistance (ASR) obtained by fitting the low frequency arc corresponds to a decrease in electrode surface exchange coefficient *k*_q_, shown in (**b**).

For example, recent computational work by Lee and Morgan has demonstrated how different transition metal cations’ redox activity and surface polarity of different compositions lead to very different surface defect chemistry and surface properties of perovskites [66]. Another example relating bulk and surface chemistry is the case where elastic and electrostatic effects within the bulk of the electrodes can contribute to the driving force for intrinsic surface segregation, where excess A-site cations enter the surface region as enrichment or secondary phases. Lee et al. (different authors) demonstrated the important roles of both elastic and electrostatic effects in A = (Sr, Ca, Ba) segregation in acceptor-doped (La, A^2+^)MnO_3_ both computationally using DFT and experimentally using analysis of thin film electrode surface morphology, chemistry, and electronic structure after high temperature anneals [67]. In this composition, as with many oxygen electrodes, some A-site cations are acceptor dopants exhibiting a relative negative charge within the lattice, so they can be electrostatically attracted to positively-charged oxygen vacancies in the surface region. Additionally, the results demonstrated the clear influence of elastic effects, as segregation was more severe the larger (more size-mismatched vs. La) the acceptor cation was, i.e., in the order Ba > Sr > Ca, and segregation was less severe when the lattice expanded in lower oxygen partial pressures, better accommodating the larger dopants. More recently, preliminary studies by Perry indicate that electrochemically measured deterioration of surface exchange coefficients (*k*_q_)—see Figure 7—occurs more rapidly for larger A-site cations even when the cation is not an acceptor dopant (i.e., isovalent substitution) within thin film (Sr,A)Ti_0.65_Fe_0.35_O_3−δ_, A = Ba, Sr, Ca. In this latter case, different mechanisms of aging among these various cations on the surface could also influence the results, in addition to the degree of segregation. Nonetheless, results such as these suggest that smaller A-site cations may thermodynamically limit segregation or deterioration of surface exchange kinetics, even though from a kinetic perspective they should be able to diffuse to the surface faster. A-site cation deficiency has also been used as a method both of intrinsic acceptor doping and surface segregation prevention [68]. However when some intrinsic or extrinsic surface poisoning is unavoidable, introduction of “gettering” species within the electrode composition is a promising alternative method to counter the negative effects of surface chemical changes on surface redox kinetics. Recent work by Zhao et al. demonstrated this approach, where La was introduced both on the surface and in the bulk of a (Ce,Pr)O_2−δ_ electrode to react with Si poisoning on the surface and restore fast oxygen exchange kinetics; a further catalytic effect of La cannot be ruled out, however [69]. Further details concerning characterization of surface chemistry in general are given in the section below.

## 5. Role of Surface Chemistry

Surface chemistry is expected to play a critical role in the oxygen surface exchange process, yet relatively more attention has in the past focused on bulk electrode chemistry, with the implicit assumption that the surface is a simple termination of the bulk, containing transition metal cations for catalytic activity. However, recent focused studies of electrode surface chemistry, taking advantage of advances in surface characterization techniques, have enabled identification of significant differences in surface and near-surface compositions vs. the bulk, particularly after or during exposure of electrode materials to typical operation temperatures [70,71,72,73].

A number of techniques have been developed to probe, in situ or in operando, the bulk and surface chemistry of oxygen electrodes during exposure to realistic operating temperature, gas atmosphere, and/or polarization conditions. Techniques include those based on X-ray diffraction [74,75,76], X-ray fluorescence [77], X-ray absorption spectroscopy [78], neutron diffraction [79] (largely for bulk information), “ambient pressure” X-ray photoelectron spectroscopy [80] and photoelectron microscopy [81], Raman spectroscopy [82], scanning probe microscopy [83], and environmental scanning or transmission electron microscopy [84]. Each of these methods may be coupled with simultaneous ac-impedance spectroscopy measurement of half or full cells to correlate the electrochemical performance and degradation of the electrodes with the changes in local chemistry and structure [79]. A comprehensive review of results from all of these techniques is beyond the scope of the present paper, and the interested reader is directed to the references mentioned herein for further information.

Among recent developments in surface analysis and ion scattering methods, ion beam-based techniques including low energy ion scattering (LEIS) and time-of-flight secondary ion mass spectrometry (SIMS) techniques have proved to be particularly useful probes of the surface composition. Beneficial features of ion beam-based techniques include: (1) shallow (surface-sensitive) information depths because ions in the relevant energy ranges do not penetrate into a solid as far as X-ray or electron beams and (2) mass-sensitivity, thus enabling identification of isotopes as well as elements. Indeed, SIMS analysis of ^18^O isotopic tracer diffusion profiles in samples having undergone anneals in gaseous ^18^O at high temperatures has been used for several decades to derive the tracer oxygen exchange coefficient (k*) and diffusivity (D*) in ionic and mixed conductors [85,86]. I^2^CNER in Kyushu University provides a unique environment for ion beam surface analysis. In this section, examples of results of surface analysis of oxygen electrodes will be briefly introduced, mainly focusing on (La,Sr)(Co,Fe)O_3_ perovskite oxide. As mentioned earlier, the composition La_0.6_Sr_0.4_Co_0.2_Fe_0.8_O_3−δ_ (LSCF), which shows good ionic and electronic conductivities [87], along with high diffusivity and surface exchange coefficients, is a popularly used oxygen electrode of intermediate temperature SOFCs [88]. This material is adopted as a model system to demonstrate ways in which LEIS and SIMS techniques can assist investigations of the relationship between surface chemistry and oxygen surface exchange kinetics.

### 5.1. Application of ^18^O Diffusion Profile Measurements by SIMS

**Figure 8:**
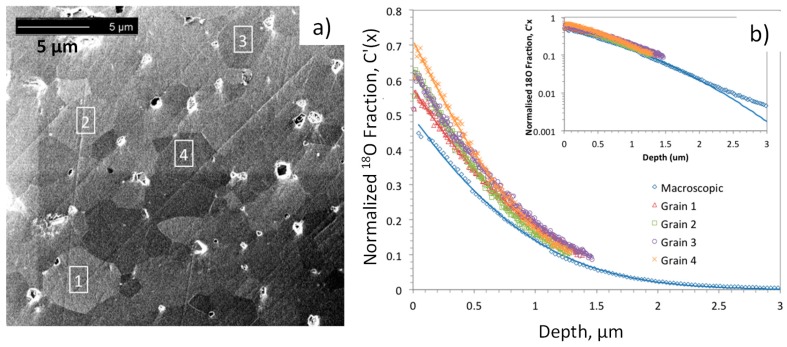
Depth profiling within individual grains in a polycrystalline LSCF sample exchanged at 450 °C in ^18^O-enriched O_2_ gas for 3721 s, using focused ion beam-secondary ion mass spectrometry (FIB-SIMS). (**a**) Microstructure, showing analyzed areas by ion-induced secondary electron image. Number labels in (**a**) identify grains from which the profiles plotted in (**b**) were obtained. Reprinted with permission of The Electrochemical Society, from [90] by Druce et al.

One question in the study of electrode surfaces is the extent to which crystal orientation or surface termination influences the surface exchange kinetics and diffusivity in the electrode, determining its performance. This question has been addressed for the highly anisotropic Ruddlesden-Popper structured electrodes by SIMS analysis of ^18^O profiles in epitaxial thin films grown with different orientations. For example, by this approach (La,Sr)_2_CoO_4_ was demonstrated by Chen et al. to exhibit faster oxygen diffusion along the ab-plane than the *c*-axis, but the surface exchange kinetics of films grown in the (001) and (100) orientations were not significantly different. The result was attributed to Sr segregation on both terminations, slowing oxygen incorporation [89]. Another approach to study this question is to take advantage of finer focusing of the SIMS incident ion beam (e.g., Ga ion gun in FIB-SIMS) to enable depth profiling within individual grains presumably exhibiting different orientations in polycrystalline ceramic samples (it should be noted that a conventional SIMS primary ion beam analyzes areas on the order of hundreds of microns, much larger than the size of typical ceramic grains or electrode particles). One limitation with this approach is that the depth that can be measured is limited to approximately the lateral dimensions of the sputtered crater, owing to loss of signal for deeper craters. An example of this method applied to a dense LSCF ceramic, with ^18^O exchange at 723 K, is shown in Figure 8, after [90] by Druce et al. While not nearly as anisotropic as the aforementioned layered structures, the perovskite LSCF exhibits slight rhombohedral distortions, and different surface terminations of different grains, having different sites and energetics for oxygen adsorption, dissociation, and incorporation may be possible. Further details of the analysis and fitting are given in [90].

**Figure 9:**
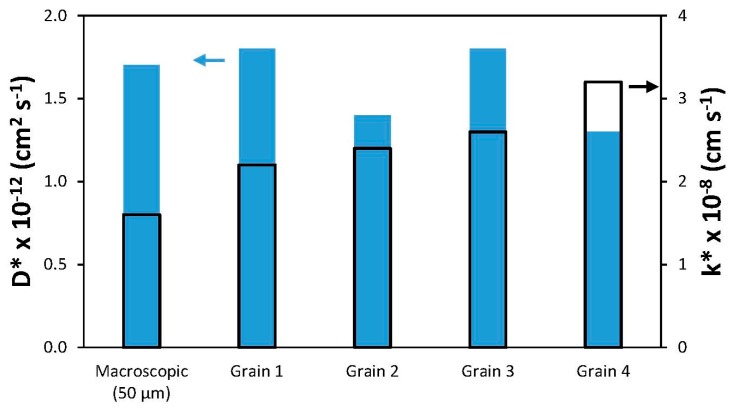
Results from the fits shown in Figure 8b. Data taken from [90] by Druce et al.

Fits to the depth profiles within the grains and a macroscopic profile are shown as solid lines in Figure 8b, and the D* and k* values extracted from these fits (from [90]) are summarized in Figure 9. While the k* values within individual grains are slightly higher than the macroscopic k* value, they and the D* values do not vary much from grain to grain. Again, similar surface chemistry as a result of Sr segregation may be a factor in this result, as discussed in the later section on LEIS.

While the results in Figure 8 and Figure 9 were obtained using a relatively high energy Ga ion beam, better depth resolution in addition to the already higher lateral resolution, may be obtained in some materials systems using Cs^+^ or C_60_^+^ sputtering [91,92].

Another question relating to electrode surface chemistry and performance is the extent to which strain state may be tuned to optimize surface exchange kinetics. Again epitaxial thin film model electrodes, such as deposited by PLD, are an asset in performing fundamental studies, as strain can be induced by the energetic growth process and/or by coherent lattice mismatch with the substrate or adjacent layers (accounting for thermal and chemical expansion). Beyond enhancements in oxide ion mobility [41] or incorporation kinetics that may be derived by stretching the lattice through which the ion passes, there is also a chemo-mechanical coupling in many systems, whereby the applied strain can lead to changes in point defect chemistry [93]. Both of these effects could, in principle, alter surface oxygen incorporation/excorporation energetics. Again, tracer ^18^O isotope diffusion profiles, measured ex situ by SIMS, as well as electrochemical impedance measurements, are providing some insight into the magnitude of the effect. For example in-plane tensile strain has been shown to accelerate both k* and D* into (100)-oriented epitaxial La_1−*x*_Sr*_x_*CoO_3−δ_ [94] and to improve *k*_q_, increase the oxygen interstitial concentration, and stabilize the surface chemistry in the anisotropic Nd_2_NiO_4+δ_ Ruddlesden-Popper phase with tensile strain along the *c*-axis [95]. Multilayer electrode structures are also the subject of research into strain effects; for example, Hyodo et al. fabricated a “laminated film” with layers of Cu and Ga-doped Pr_2_NiO_4_ (PNCG) and Sm-doped CeO_2_ (SDC), where the former phase was expected to be in compression and the latter in tension on the basis of lattice parameters [96]. Using ^18^O tracer diffusion, the results suggested that mechanical strain has a large influence on the oxygen diffusivity; however, the enrichment in surface ^18^O concentration was more significant. Therefore, surface activity seemed to be more sensitively affected by mechanical strain effects in that system.

### 5.2. Evaluation of Surface Chemistry by LEIS

**Figure 10:**
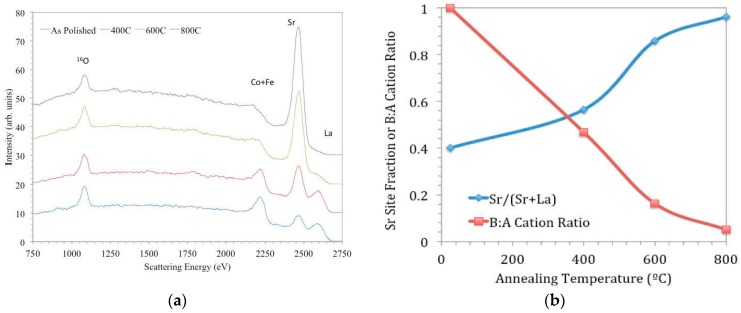
(**a**) LEIS spectra using 3 keV ^4^He^+^ primary beam for LSCF ceramics annealed for 8 h at various temperatures. Adapted from [97] by Druce et al. with permission from Elsevier; (**b**) Fraction of Sr on A-site and B:A cation ratio quantified from spectra in (**a**).

LEIS measurements are able to identify elements in the outermost atomic monolayer, and this extreme surface sensitivity is of interest for understanding the composition immediately in contact with gaseous oxygen during the exchange process. Like SIMS, LEIS is often performed ex situ, where it can be applied to study electrode materials after treatment in high temperatures and gas atmospheres typical of operation conditions [70,97,98,99,100]. While one expects qualitative similarities in the ex situ results on polycrystalline, dense ceramics or thin films and the surface chemistry of real, porous ceramic electrodes in operando, some differences owing to the microstructure and environment changes (e.g., surface-to-volume ratio, surface curvature, grain boundary density, impurity gas concentration, impact of adjacent cell layers, etc.) may be possible. In this regard, an additional advantage of LEIS is that there are few limitations in the form of the sample; real porous electrodes may be studied as well as model systems such as dense ceramics or thin films. One outcome of the model sample, ex situ studies has been the confirmation of A-site cation termination/segregation widely observed in the SOFC/SOEC/catalysis community using a variety of other approaches that have slightly less surface sensitivity, such as total reflection X-ray fluorescence, nanoprobe Auger spectroscopy, SIMS, scanning electron microscopy with energy-dispersive spectroscopy, and X-ray photoelectron spectroscopy [73,101,102,103,104]. For example, Figure 10, taken from [97], shows a series of LEIS spectra of LSCF ceramic samples; one has been measured after polishing, and the others were measured after annealing for 8 h at different temperatures.

In Figure 10a, the peaks in the spectrum for the as-polished sample correspond to all the elements present in the bulk composition—O (1181 eV), Fe (2311 eV) and Co (2342 eV; these cannot be resolved with the He beam due to similar masses), Sr (2541 eV), and La (2701 eV). (Peak energies quoted correspond to the high energy onset of the peaks, and the peak apexes appear at slightly lower energies due to inelastic scattering processes.) Peak intensities for surface elements are proportional to their coverage (by a sensitivity factor dependent on the element’s mass), and so changes in their intensities are indicative of changes in outermost surface coverage. From the other spectra in Figure 10a, it can be seen that with progressively higher annealing temperatures, the coverage of Sr increases at the expense of those of La and the transition metal cations. In Figure 10b, quantified peak areas from Figure 10a are plotted to show the evolution of surface coverage in terms of cation ratios for given annealing temperatures. The increase of Sr coverage is most pronounced between 673 and 873 K under these conditions; this result helps to explain the relative insensitivity of k* to LSCF grain orientation discussed earlier, since that sample was at 723 K for the ^18^O exchange.

**Figure 11:**
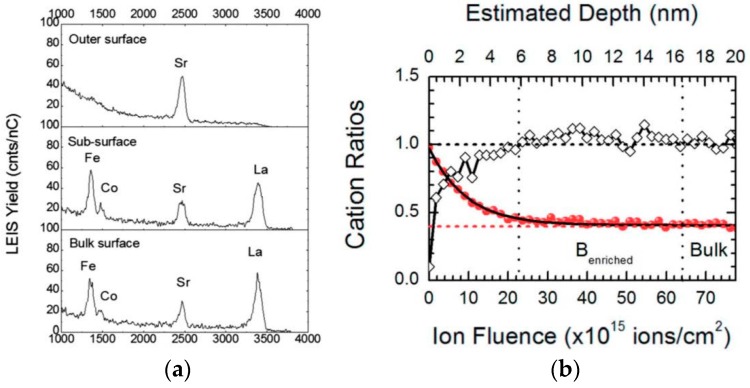
LEIS measurements with ^20^Ne^+^ primary beam at 6 keV at different depths of an LSCF ceramic annealed for 12 h at 1273 K. (**a**) Selected partial spectra corresponding to the very outer surface, the sub-surface and “bulk” regions; (**b**) Depth profile of Sr site fraction (solid circles) and B:A cation ratio (open diamonds). Reproduced from [98] by Druce et al. with permission of The Royal Society of Chemistry.

A sequence of LEIS spectra can also be obtained after stepwise removal of material by low energy (500 eV) Ar ion sputtering to provide depth profiles of elements. By this approach, one may, for example, study not only the outer monolayer chemistry but also the sub-surface region after Sr surface enrichment/segregation has occurred. Figure 11 (from [98]) shows sample LEIS spectra (partial energy range) at different depths and a resulting depth profile measured on a LSCF ceramic sample after annealing for 12 h at 1273 K.

While the outermost surface is characterized by an absence of La or transition metal cations in Figure 11a (though there is evidence for sub-surface La indicated by the rise in background signal below 3500 eV), the La, Co, and Fe peaks appear in the outer monolayer analyzed after removal of some material. The depth profile in Figure 11b demonstrates not only the B-site deficient surface region but also a sub-surface region slightly enriched in the B-site cations. This enrichment was shown to be more pronounced in GdBaCo_2_O_5+δ_ (GBCO) and La_2_NiO_4+δ_ (LNO) [98], which also lacked transition metal cations in the initial outer monolayer. Interestingly, various oxide structures (perovskite, double perovskite, Ruddlesden-Popper, fluorite) and chemistries exhibit surfaces enriched with rare earth or alkaline earth cations with larger ionic size and smaller oxidation number than their hosts. As discussed earlier in the section discussing the relationship between bulk and surface chemistry, this surface enrichment phenomenon may in principle be explained by a combination of elastic and electrostatic effects with possible influence from extrinsic factors, such as gas atmosphere. It should also be noted that while these particular LEIS measurements have been performed on ceramic pellets after heat treatments, they may also be carried out on electrochemical cells after testing, to provide some insight into the impact of surface chemistry on actual electrode performance, particularly oxygen surface exchange rate.

**Figure 12:**
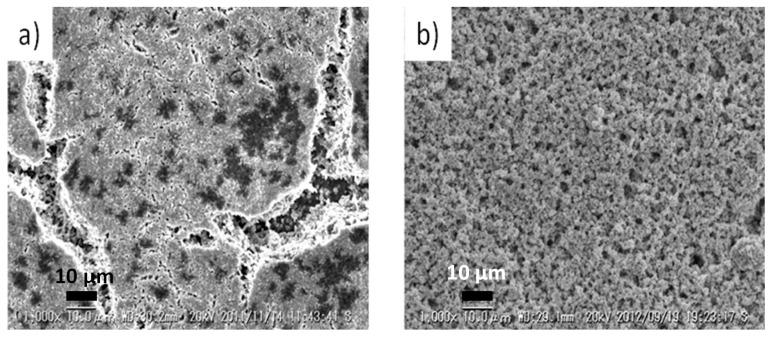
Scanning electron microscopy (SEM) images of LSCF cathodes exposed to different concentrations of sulfur impurity in the gas stream during electrochemical measurements of the degradation process, (**a**) 1513 ppm S; (**b**) 752 ppm S. Taken with permission from Elsevier, from [105] by Xie et al.

Finally, surface chemistry studies by other methods have also indicated that the intrinsic segregation phenomena may interact with extrinsic poisoning effects to alter surface chemistry. A common impurity in the gas stream for the SOFC cathode is S. Although the aforementioned LSCF composition is now popularly used as the oxygen electrode of SOFCs and SOECs, this oxide is sensitive to the presence of the S impurity, with which it reacts, resulting in the electrode surface deactivation. Figure 12 shows scanning electron microscopy (SEM) images of LSCF electrodes having been exposed to impurity sulfur during power generation and degradation measurements [105]. Evidently, regions appearing to have partially melted, where high amounts of S and Sr are detected, are observed (these are the darker spots in Figure 12a). Therefore, it appears that surface segregated Sr may easily react with SO_2_ in the air to form SrSO_4_ which easily sinters and blocks the surface from participating in efficient oxygen reduction. Therefore, one reason for degradation of oxygen electrodes is caused by a combination of intrinsic surface segregation of Sr and extrinsic S poisoning, and this process appears relatively acute for LSCF.

### 5.3. Modeling of Oxygen Dissociation on Surfaces Absent of Transition Metal Cations

In light of the emerging experimental evidence among the ionics community of A-site segregation and AO termination in perovskites and related structures under typical SOFC/SOEC operating conditions, computational simulations have been applied to understand the mechanism of oxygen exchange on surfaces absent of transition metal cations. Density functional theory calculations can estimate the energy of various configurations of an electrode slab interacting with oxygen, to determine, for example, the lowest energy oxygen adsorption site(s), whether those configurations indicate associated or dissociated oxygen, and whether such a process is energetically spontaneous. In addition, transition state analysis by nudged elastic band calculations can indicate the activation energy barriers for moving between one step in the reaction to the next. These approaches have recently been applied to study oxygen chemisorption and dissociation on SrO-terminated SrTiO_3_ and LaO-terminated La_2_NiO_4_, both for the stoichiometric and oxygen-deficient compounds, where oxygen vacancies are introduced via ionically compensated acceptor doping with Fe in the case of SrTiO_3_. Details of the calculation approaches are given elsewhere [106,107]. Both oxygen chemisorption and dissociation may be enabled by charge transfer to the oxygen, so the presence of accessible electron density in or near the surface can help these processes to occur. In both SrO-terminated SrTiO_3_ and LaO-terminated La_2_NiO_4_, the presence of oxygen vacancies near the surface was shown to facilitate (lower the energy barrier for) oxygen adsorption and dissociation on the surfaces, by enabling charge transfer. In the case of SrO terminated stoichiometric SrTiO_3_, oxygen chemisorption and dissociation was not energetically favorable, since the closed-shell configuration of Sr^2+^ does not contribute electron density that would interact with chemisorbed oxygen. For LaO-terminated stoichiometric La_2_NiO_4_, however, oxygen chemisorption was shown to be exothermic (−0.73 eV and −0.59 eV for chemisorption on a slip position and La-La bridge position, respectively), because the extra 5d electron in La^3+^ can polarize its 6s valence orbitals and enable charge transfer from the surface, destabilizing the oxygen molecule. Calculated energetics for dissociation of oxygen after chemisorption on the aforementioned surfaces are summarized in Table 1.

## 6. Conclusions

### 6.1. Summary

Oxygen electrodes, typically mixed conducting perovskites and related structures, play a vital role in impacting the efficiency and lifetime of SOFCs/SOECs as the location of electrochemical oxygen incorporation or evolution. They need to demonstrate excellent catalytic activity for rapid surface oxygen exchange, good bulk transport properties (electronic and ionic), and maintain thermo-chemo-mechanical stability in contact with other cell components and often impure gas atmospheres for multiple years of operation including start-stop cycling. In this non-exhaustive review we have highlighted some of the work at I^2^CNER, Kyushu University, set in the context of work in the broader community, which seeks to understand the roles of bulk and surface chemistry in these aspects of oxygen electrode performance. Understanding the impact of bulk composition on carrier concentrations and mobilities, surface exchange kinetics, and chemical expansion coefficients remains an active area of research, both experimentally and computationally. At the same time, an understanding of the relationship between bulk and surface chemistry is being developed through computational simulations and experimental high resolution and/or operando surface chemistry studies, that may assist design of electrodes with more robust surface chemistries that are impurity tolerant or do not show rapid surface segregation. As cross-cutting approaches, the use of strain and/or a high density of active interfaces show promise for enhancing bulk transport and surface exchange kinetics.

### 6.2. Outlook

Although fuel cells using solid ceramic electrolytes have been in development for over 60 years, there remain significant opportunities for improving the efficiency and durability of oxygen electrodes in these SOFCs and in the newer SOEC technologies.

Concerning bulk chemistry, the area of “electro-chemo-mechanics”, i.e., the coupling between mechanical, chemical, and electrical states of materials, is an emerging research theme underlying electrode performance improvements, including (1) enhancements in transport and surface reactivity realized by tailoring strain state (mechano-electrical and mechano-electrochemical coupling) as well as (2) mitigation of deleterious chemical expansion during operation, induced by stoichiometry changes (chemo-mechanical coupling). In the case of intentionally applied strain (1), an understanding of how to realize high levels of durable strain in the appropriate direction(s) in real electrodes under operating conditions should be pursued, and an understanding of what magnitude of enhancements in oxide ion mobility, non-stoichiometry, and surface exchange kinetics can be accomplished realistically in devices, given the modest strain levels that can be realized in brittle ceramics, should be developed. At a fundamental level, partial understanding of oxygen mobility in perovskites has been developed, but further insight into how cation polarizability, ionic radii, tolerance factor, free volume, lattice strain, interfacial effects, and other factors can be optimized for the fastest possible ionic mobility should be developed in the future. Concerning chemical expansion (2), further insight into factors impacting the magnitude of coefficients of chemical expansion is needed, and modeling of chemical stress development spanning atomic to device length scales would help to mitigate these stresses and maximize device mechanical integrity for high durability.

Regarding surface chemistry and oxygen surface exchange kinetics, areas of particular interest are: (1) clarifying rate-limiting steps and mechanisms of oxygen incorporation/excorporation with atomistic insight both experimentally and computationally; (2) exploiting the unique properties of hetero-interfaces, grain boundaries, and other long range defects intersecting the surface; (3) identifying the theoretical optimal composition(s) for the outermost atomic monolayers; and (4) learning how to control the outermost chemistry in operating conditions via bulk and surface chemical tailoring. Each of those areas is particularly strategic because the high activation energy of the oxygen exchange process limits the operating temperature range of the devices at present and can dominate efficiency losses at lower temperatures. For practical use of SOFCs/SOECs, an additionally important challenge is increasing the long-term stability. Therefore many research efforts are aimed at identifying, understanding, and addressing degradation mechanisms that lead to increases in internal resistances and overpotentials during operation. For example, learning how to control and mitigate electrode surface poisoning, either by intrinsic large cation segregation or by extrinsic species depositing or reacting with the surface is a particularly significant area of research along these lines. Overall, oxygen electrode development remains a very active field, and continued effort to understand fundamental structure-property relationships in both the bulk and surface of electrodes is needed for development of descriptors for rational design and discovery of superior electrode chemistries.

## Figures and Tables

**Table 1:** Calculated Energy of Oxygen Dissociation on Simulated Surfaces (lowest energy configurations) vs. the Chemisorbed State, from [106,107] by Akbay et al. and Staykov et al.

State	SrO-Terminated SrTiO_3_		LaO-Terminated La_2_NiO_4_	
	Defect Free	With V_O_ (and Fe)	Defect Free	With V_O_
Transition barrier for dissociation	-	0.50 eV (iii)	1.35 eV (i) 1.10 eV (ii)	0.28 eV
Dissociated state (vs. chemisorbed state)	3.4 eV (not stable)	−2.38 eV	1.10 eV (i) 0.56 eV (ii)	−0.93 eV

(i) Chemisorption on slip position and dissociation on La-La bridge site; (ii) Chemisorption on La-La bridge position and dissociation on La-La bridge site (wider O separation); (iii) Dissociation into nearby surface oxygen vacancies; requires 1.02 eV for surface V_O_ migration to be in that configuration, so surface oxygen migration becomes rate-limiting.
